# A Giant Exophytic Gastric GIST Mimicking Ovarian Cancer: A Diagnostic Pitfall on CT and [^18^F]FDG PET/CT

**DOI:** 10.3390/diagnostics16111575

**Published:** 2026-05-22

**Authors:** Sang Jun Byun, Sun-Jae Lee, Byungwook Choi

**Affiliations:** 1 Department of Radiation Oncology, Keimyung University School of Medicine, Daegu 42601, Republic of Korea; kryph@dsmc.or.kr; 2 Department of Pathology, School of Medicine, Daegu Catholic University, Daegu 42472, Republic of Korea; pathosjlee@cu.ac.kr; 3 Department of Nuclear Medicine, School of Medicine, Daegu Catholic University, Daegu 42472, Republic of Korea

**Keywords:** gastrointestinal stromal tumor, computed tomography, [^18^F]FDG PET/CT, pelvic mass, ovarian neoplasms, differential diagnosis

## Abstract

A 66-year-old woman was referred for evaluation of a large pelvic mass suspected to be ovarian cancer. Contrast-enhanced computed tomography (CECT) revealed a giant multiseptated cystic pelvic mass with enhancing solid components; although its superior aspect closely abutted the gastric serosa, its predominant pelvic location raised concern for an adnexal malignancy. Subsequent [^18^F]fluorodeoxyglucose positron emission tomography/computed tomography ([^18^F]FDG PET/CT) demonstrated mild uptake confined to the viable solid portion (SUVmax 2.72) without hypermetabolic nodal or distant metastases. Exploratory laparotomy revealed a giant pedunculated tumor arising from the gastric antrum and descending into the pelvis. Histopathology confirmed an epithelioid gastrointestinal stromal tumor positive for CD117, DOG1, and CD34. This case highlights an important diagnostic pitfall in which a giant exophytic gastric GIST may mimic ovarian cancer because of its pelvic location and cystic-solid appearance. Careful correlation of CECT, fused [^18^F]FDG PET/CT, and pathologic findings is essential for accurate assessment of the organ of origin in large abdominopelvic masses.

**Figure 1:**
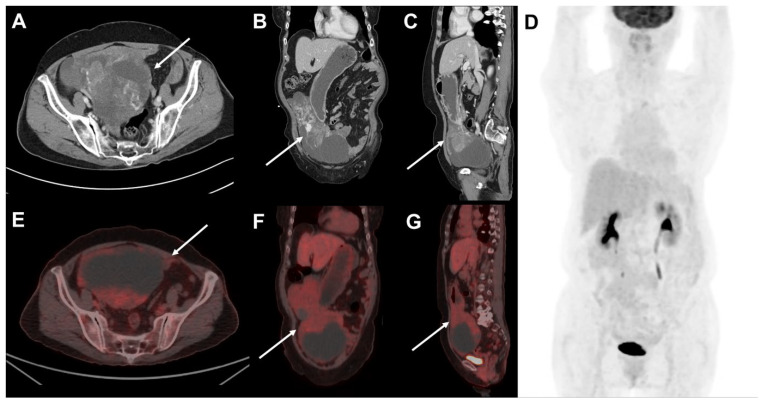
A 66-year-old Korean woman was referred to our hospital for evaluation of a large pelvic mass initially suspected to represent an ovarian tumor on pelvic ultrasonography performed at a local clinic. Contrast-enhanced computed tomography (CECT) was performed first. CECT images in (**A**) axial, (**B**) coronal, and (**C**) sagittal planes demonstrate a huge, predominantly multiseptated cystic pelvic mass with heterogeneously enhancing solid components. On preoperative CECT, there was no definite imaging evidence of bowel-loop encasement, bowel obstruction, or direct bowel invasion. The superior margin of the mass was in close contact with the gastric antrum (arrows in (**A**–**C**,**E**–**G**)); however, because the dominant tumor bulk was located in the pelvis and the lesion showed a multiloculated cystic-solid morphology, this relationship was initially interpreted as nonspecific abutment or compression rather than a definite gastric pedicle. The unusual elongated and caudally displaced appearance of the stomach on coronal and sagittal CECT images was not considered a separate anatomic variant or postsurgical change, as no history of gastric surgery or known concomitant gastric disease was documented in the available clinical records. In retrospect, this configuration was attributed to traction and mass effect caused by the pedunculated antral tumor descending into the pelvis. Subsequent [^18^F]fluorodeoxyglucose positron emission tomography/computed tomography ([^18^F]FDG PET/CT) was then performed. (**D**) The maximum intensity projection image shows mild [^18^F]FDG uptake localized within the pelvic mass without hypermetabolic nodal or distant metastatic disease. Fused [^18^F]FDG PET/CT images in (**E**) axial, (**F**) coronal, and (**G**) sagittal planes demonstrate mild uptake confined to the viable solid portion of the tumor (SUVmax 2.72) and facilitate anatomic correlation with the CECT findings. Pelvic MRI was not performed before surgery. Based on outside pelvic ultrasonography and CECT, no specific histologic subtype of ovarian tumor was definitively assigned preoperatively; however, the imaging-based differential diagnosis favored a primary cystic epithelial ovarian neoplasm, particularly a mucinous borderline or malignant ovarian tumor. Other relevant differential diagnoses for a large cystic-solid pelvic mass on CECT and [^18^F]FDG PET/CT include metastatic ovarian tumors, degenerated subserosal uterine leiomyomas, tubo-ovarian inflammatory masses, and peritoneal or mesenteric soft-tissue tumors. Unlike typical gastric GISTs that present as intramural masses, pedunculated or extraluminal gastric GISTs may descend into the pelvis and mimic gynecologic malignancies because of their predominant pelvic location and cystic-solid appearance [1,2,3,4]. A recent systematic review also emphasized that GISTs may be misinterpreted as primary ovarian tumors or may involve the ovaries, underscoring the importance of careful assessment of the organ of origin in large pelvic masses [4]. In such cases, CECT with multiplanar reconstruction may provide the most important clue to tumor origin by demonstrating a pedicle or site of attachment to the stomach [1,5], whereas fused [^18^F]FDG PET/CT offers complementary information by showing metabolic activity in the viable solid portion and excluding overt nodal or distant metastatic disease [3]. Giant GISTs may show deceptively mild or heterogeneous [^18^F]FDG uptake because of extensive necrosis and cystic degeneration despite clinically significant disease [3]. In the present case, the relatively low SUVmax of 2.72 despite the large tumor size was considered to reflect the predominance of cystic degeneration and ischemic necrosis, which reduced the proportion of metabolically active viable tumor tissue. Therefore, [^18^F]FDG uptake was mainly confined to the residual viable solid component rather than the extensive necrotic or cystic portions of the mass. Retrospective review of the preoperative CECT images, performed after the intraoperative findings were established, confirmed that the gastric contact corresponded to the site of pedunculated antral attachment.

**Figure 2:**
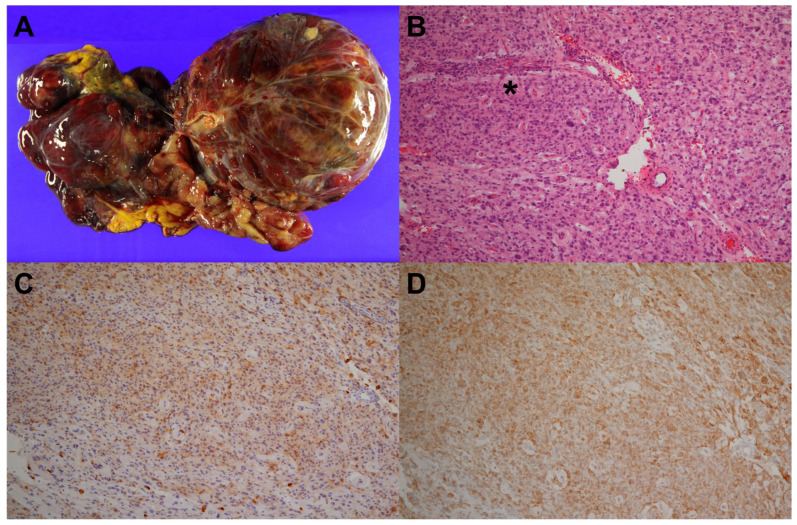
Surgical exploration was subsequently performed to establish a definitive diagnosis. (**A**) Intraoperative findings revealed that the bilateral ovaries were normal. Instead, a giant, well-encapsulated tumor measuring 21.5 cm × 10.0 cm × 2.5 cm was found arising from the anterior wall of the gastric antrum and extending into the pelvis via a stalk, with no other remarkable intraoperative findings. (**B**) Microscopic examination (H&E stain, ×100) demonstrates solid sheets of predominantly epithelioid tumor cells (asterisk) with scattered pleomorphic multinucleated giant tumor cells. Immunohistochemical staining shows diffuse strong positivity for CD117 (c-kit) (**C**) and DOG1 (**D**). The tumor was also positive for CD34. The Ki-67 labeling index was approximately 5–10% in the viable tumor areas. Taken together with the imaging findings in Figure 1, the operative and pathologic findings established the diagnosis of an epithelioid gastric GIST with pedunculated extraluminal growth. This case underscores the importance of correlating cross-sectional imaging, fused [^18^F]FDG PET/CT, intraoperative findings, and histopathology to avoid misclassification of the organ of origin in large abdominopelvic masses.

## Data Availability

The data presented in this study are available on request from the corresponding author due to patient privacy constraints.
